# Water-Floating Hydrogel Polymer Microsphere Composites for Application in Hydrological Mining of Cu(II) Ions

**DOI:** 10.3390/nano13192619

**Published:** 2023-09-22

**Authors:** Andrei Honciuc, Ana-Maria Solonaru, Mirela Honciuc

**Affiliations:** “Petru Poni” Institute of Macromolecular Chemistry, Gr. Ghica Voda Alley 41A, 700487 Iasi, Romania; solonaru.anamaria@icmpp.ro

**Keywords:** metal ion extraction and recovery, hydrological mining, polymer adsorbents, Pickering emulsion polymerization technology, hydrogel polymer composites

## Abstract

Innovative materials and technologies capable of extraction and recovery of technologically relevant metal ions from various water sources, such as lakes, oceans, ponds, or wastewater reservoirs, are in great demand. Polymer beads are among the most well-known solid-phase adsorbents and ion exchangers employed in metal ion recovery. On the other hand, hydrogels are an emerging platform for producing innovative adsorbents, which are environmentally friendly and biocompatible materials. In this work, we take advantage of both technologies and produce a new type of material by loading nanostructured polymer microsphere adsorbent into a PVA matrix to obtain a hydrogel polymer microsphere (HPM) composite in the form of a block. The main role of the poly(4-vinylpyrridine-co-methacrylic acid) microspheres is to adsorb metal ions, such as Cu(II), from model water samples. The secondary role of these microspheres in the hydrogel is to change the hydrogel morphology by softening it and stabilizing it under a foam-like morphology. The foam-like morphology endows these composites with the capability of floating on water surfaces. In this work, we report, for the first time, an HPM composite capable of floating on water surfaces and extracting Cu(II) ions from model water samples. This could enable more environmentally friendly hydrological mining technologies by simply deploying adsorbents on water surfaces for metal ion extraction and recovery, thus eliminating the need for water pumping and mechanical processing steps.

## 1. Introduction

Water and aqueous reserves from any source, such as oceans, lakes, or used waters from domestic, industrial, commercial, or agricultural activities, can be a valuable secondary resource for raw materials [1,2]. Innovative materials and technologies that can be deployed in hydrological mining aimed at recovering technologically relevant metal ions are in great demand. Synergistically, the same materials and technologies can be deployed for the removal of toxic metal ions from contaminated wastewater. However, recovery of metal ions must also be economically feasible; thus, materials and technologies that rely on minimal energy consumption are desirable. The current materials and technologies for wastewater treatment and purification have been extensively reviewed [3,4,5]. Thus far, solid-phase adsorbents and ion exchangers are deployed on a large scale for wastewater treatment and purification [4]. These could be cheap adsorbents that come from agricultural waste, food waste, biomass, inorganic materials, natural or synthetic polymers, etc. [6,7,8]. Among these, engineered nanomaterials could play a significant role [3,4,9], for example, micro or nanostructured polymers, such as microporous monoliths [10]. Recently, Pickering emulsion technology [10,11] and hydrogel-based composites [12] have been considered viable green water-based platforms for the preparation of solid- and semi-solid-phase adsorbents for the removal of metal ions from water and soil. As already alluded, future solid-phase adsorbent materials must also address the issue of energy efficiency and play the role of enablers for green technologies. For example, one way the energy consumption can be decreased is to eliminate the need for the energy-intensive process of pumping water through columns filled with ion exchangers. Instead, water-floating materials could be deployed on the surface of waters and ponds to adsorb polluting heavy metal ions or organic pollutants. Currently, such innovative materials with self-floating capabilities are being developed, among which we mention functionalized hollow glass microspheres [13,14], chitosan-based aerogels [15], graphene oxide aerogels [16,17], self-separating polymers [18], covalent-organic frameworks (COFs) [19], etc. On the other hand, the synthesis of hydrogels as adsorbents has been reported with good adsorption capacities [7,12,20,21]. Unfortunately, none of these studies focus on developing water-floating hydrogel or hydrogel polymer composites for the metal-ion extractions from water. Water-floating capability can be a great advantage for novel technologies in hydrological mining, as it minimizes the need for pumping large amounts of water into ion-exchange columns or other energy-intensive technologies requiring a great number of mechanical operations. In this work, we address exactly this aspect, and we show that such materials can be prepared and deployed in the extraction and recovery of some technologically relevant metal ions, such as Cu(II) metal ions [6]. The material consists of polymer microspheres synthesized via Pickering emulsion polymerization technology (PEmPTech) [22]. Due to the unique surface nanostructuring with the silica nanoparticles, these microspheres are perfectly dispersible in aqueous solutions and hydrophilic hydrogel precursor solutions, thus enabling their utilization in hydrogel matrices. Thus, we take advantage of both technologies and produce a new type of material by loading nanostructured polymer microsphere adsorbents into a PVA/Glycerol hydrogel to obtain a hydrogel polymer microsphere (HPM) composite in the form of a block. Upon inclusion in the hydrogel, these polymer microspheres can aid in foaming the hydrogel and stabilizing this foam, endowing the composite with a solid foam-like structure and water-floating capabilities. Therefore, we have prepared two classes of HPM composites: (i) hydrogel polymer microsphere blocks (HAM) and (ii) hydrogel polymer microsphere foams that can float on the surface of water (FAM). These were then deployed for the extraction and recovery of Cu(II) ions from model water samples. From a mass transfer perspective, materials that float on the surface may exhibit different ion adsorption capacities than those that are completely submersible in water. Therefore, in this work, we analyze, for the first time, this aspect of the capacity of extraction of metal ions between adsorbents that float and those that are completely submersible in water.

## 2. Materials and Methods

### 2.1. Materials

Tetraethylorthosilicate (TEOS) 99%, (3-glycidoxypropyl)trimethoxysilane (Gly) 98%, divinylbenzene (DVB) technical grade 80%, containing monomethyl ether hydroquinone as inhibitor, 4-vinylpiridine (4-VP) 95%, containing 100 ppm hydroquinone as inhibitor, methacrylic acid, (MA) 99% stabilized with 250 ppm 4-methoxyphenol, aluminium oxide (Al_2_O_3_), and 2,2′-Azobis(2-methylpropionitrile) (AIBN) 98% were purchased from Sigma-Aldrich (Merck, KGaA, Darmstadt, Germany). Poly(vinyl alcohol) (PVA) granules with an average molecular weight (Mw) of 12.4 × 10^4^ g/mol and a 99–100% degree of hydrolysis and Glycerol (Gly) (99.6%) were purchased from Acros. Organics (Geel, Belgium). Copper chloride (II) dihydrate pure p.a. (CuCl_2_·2H_2_O) was purchased from Chempur (GmbH Rueppurrer, Karlsruhe, Germany); hydrochloric acid (HCl) ≥ 37% was purchased from Fluka (Honeywell Specialty Chemicals, Seelze, Germany); ethanol absolute (EtOH), 99.3%, toluene, and n-hexane were purchased from Chemical Company; and ammonium hydroxide solution (28–30%) was purchased from analysis EMSURE ACS. Reag. Ph Eur. Supelco. All reagent-containing inhibitors were passed through aluminium oxide to remove the stabilizer before usage. All the aqueous solutions were prepared in freshly distilled water.

### 2.2. Synthesis and Functionalization of Silica Nanoparticles and Polymer Microspheres

The preparation procedure for silica nanoparticles (NP) and silica nanoparticles functionalized with epoxy (NP-Gly) by reaction with Gly was previously reported [23]. Briefly, in a 1000 mL round-bottom flask, 9 mL TEOS, 300 mL EtOH, 33 mL H_2_O, and 27.7 mL NH_4_OH were mixed at room temperature and 1000 rpm. Next, 54 mL TEOS dissolved in 200 mL EtOH was slowly added via separatory funnel (for 3 h), and the final reaction mixture was left for 24 h at room temperature. After this, the mixture was neutralized with 18 mL of HCl. The obtained nanoparticles were separated by centrifugation and washed three times with EtOH and three times with water. The surface functionalization reaction proceeded by dispersing 1.2 g of silica NPs in 30 mL of EtOH and then pouring in a 250 mL flask containing 10 mL EtOH, which was purged under Ar atmosphere. The reaction mixture was stirred at 1000 rpm. Subsequently, 2 mL of Gly was added dropwise. At the end of the addition time, the reaction mixture was heated and maintained to 60 °C for 24 h. The functionalized nanoparticles were washed three times with EtOH and another three times with water before being finally redispersed in water.

For the preparation of the polymer microspheres (PMs) via PEmPTech, two vinyl-bearing monomers, 4-VP and MA, having different polarities, and DVB as crosslinker, were used for the preparation of three batches of Pickering emulsions, PM1, PM2, and PM3. These were produced by first adding 30 mg of AIBN radical initiator to a 20 mL glass scintillator vial, followed by 2.5 mL of equimolar mixture of monomers (MA or 4-VP), 0.5 mL of crosslinker (DVB), and 0.75 mL of porogen solvent—and in our case, toluene. Next, 5 mg of colloidal particles NP-Gly and 12 mL of water were added. The glass scintillator vials with Pickering emulsion were then sonicated with a Vortex mixer LLG (Lab Logistics Group GmbH, Meckenheim, Germany) for 60 s at 3000 rpm, and every Pickering emulsion was then polymerized in an oil bath for 24 h at 70 °C. After the polymerization, the products were filtered and thoroughly washed with ethanol to remove the unreacted monomers and were dried at room temperature.

### 2.3. Preparation of Hydrogel—Polymer Microspheres Composites

First, a homogenous PVA solution with a concentration of 3% was prepared by dissolving the required amount of polymer in distilled water at 90 °C and vigorously stirring for 3 h. After the polymer was completely dissolved, Glycerol was added to obtain a mixture of 1/2 ratio of PVA/Glycerol and further stirred until complete homogenization. The HPM composites were prepared by mixing the previously obtained polymer solution with different quantities of PMs (i.e., 0.45 g and 1 g) so that they resulted in samples with two ratios of PVA/PMs. These were stirred for five minutes, with 200 rpm, at room temperature, and after that, were subjected to 10 subsequent cycles of freezing (at −20 °C) and thawing (at room temperature for 8 h). Samples thus obtained were named HAM-1 and HAM-2, respectively. A different series of samples (i.e., FAM-1 and FAM-2, respectively) were prepared by following the same recipes as for HAM-1 and HAM-2, with the difference that after obtaining the solutions, these were foamed and immediately frozen in liquid nitrogen, then subjected to 10 freezing/thawing cycles. Also, a blank sample (without PMs) of PVA/Glycerol was obtained by 10 freezing/thawing cycles.

### 2.4. Measurement of Ion Extraction and Recovery Capacity of HPM Composite

The Cu(II) ion concentration in the diluted supernatant and filtrate were analyzed using a UV–vis spectrophotometer (DLAB Scientific Co., Ltd., Beijing, China). First, calibration curves were generated corresponding to maximum absorption wavelength λ_max_ = 810 nm for CuCl_2_·2H_2_O; see Figure S1 in the Supplementary Materials.

For the ion extraction, which refers to the extraction of metal ions from a stock solution, a weighted amount of HPM was immersed in 100 mL stock solution with a 5 × 10^−2^ M concentration.

The metal ion extraction capacity *q*_e_ (mg/g) was calculated with the formula:

(1) $$q_{e}=\frac{\left(c_{i}-c_{e}\right)\;V}{m_{P}}$$

where *c*_i_ (mg/L) is the initial concentration of a stock solution or the contact solution, *c*_e_ (mg/L) is the extracted concentration, *V* (L) is the volume of the sample, typically 100 mL, and *m*_P_ (g) is the dry mass of the HPM (see Table S1).

For the ion recovery, which refers to the recovery of metal ions from the polymer adsorbent, the HPM composite was immersed in 50 mL of 5% HCl. The samples were then left in this condition for approx. 12 h. Supernatant and filtrate were analyzed using UV–vis, and the metal ion recovery capacity *q*_r_ (mg/g) was calculated by

(2) $$q_{r}=\frac{c_{r}\;V}{m_{P}}$$

where *c*_r_ (mg/L) is the concentration of metal ions recovered from the HPM composite, *V* (L) is the volume of the sample, and *m*_P_ (mg) is the dry mass of the HPM composite (see Table S1).

The procedure of extraction–recovery was repeated five times unless otherwise specified.

### 2.5. Material Characterization

#### 2.5.1. Scanning Electron Microscopy

The materials were investigated with a Verios G4 UC (Thermo Fischer Scientific Inc., Eindhoven, The Netherlands) scanning electron microscope (SEM), with a 5 keV beam energy, using an Everhart–Thornley detector, beam spot 50 pA.

#### 2.5.2. Optical Microscopy

Microspheres and HPM composites were characterized with an IM-5FLD inverted fluorescence microscope (Optika Srl, Ponteranica, Italy) equipped with (i) an 8W XLED illumination source for sample analysis under transmitted light; (ii) 5W LED excitation illumination sources at 470, 560, and 385 nm and blue, green, and UV filter sets for sample analysis in fluorescence mode; (iii) color digital Camera Optika C-P6, 6.3 MP; and (iv) OPTIKA PRO VIEW (Optika Srl, Ponteranica, Italy) software for image acquisition and processing. Samples were characterized with 10× magnification objectives in transmitted illumination mode.

#### 2.5.3. Contact Angle—Washburn Method

Water contact angle of the nanostructured microspheres obtained by PEmPTech was determined via the capillary rise method, using the DCAT 15 Tensiometer balance (DataPhysics Instruments GmbH, Filderstadt, Germany), equipped with the DCATS 32 software module for calculating the contact angle via Washburn method. The capillary constant of the PM samples packed in the glass capillary was first determined using hexane. For comparison, the contact angles of marine sand, which was sieved to a granulation of <250 μm and calcinated at 850 °C, were also measured. After the determination of the capillary constant, the polymer microsphere was loaded in special glass capillaries with a porous glass bottom (DataPhysics Instruments GmbH, Filderstadt, Germany), with an inner diameter of 9 mm, outer diameter of 11 mm, and height of 62 mm, and was filled with powder up to 22.5 mL dry volume. After filling with powder, the capillary was gently knocked with a wooden popsicle stick to achieve a compact packing of the powder and removal of the packing voids. Then, the Washburn capillary was lowered gently until it touched the water surface; once it touched the water surface, the capillary stopped, and the water started rising into the capillary packed with powder. The raw data consisted of the recorded weight of the water intake vs. time. The total duration of the experiment was 40 s.

#### 2.5.4. Penetration Experiments

Penetration experiments of the HPM composites were conducted with a DCAT 15 Tensiometer balance (DataPhysics Instruments GmbH, Filderstadt, Germany), equipped with the DCATS 35 software module for penetration experiments. The penetration experiments were conducted with a metal penetration cone as the penetrometer probe. Upon free hanging of the metal cone from the piezoelectric weighing sensor, the tensiometer balance registered 20 g, and the software automatically tarred to zero before the penetration experiments. This cone was lowered slowly onto the soft HPM composite, and upon contacting the HPM surface, a negative weight due to the opposing force was recorded, which increased in absolute value with the cone immersion depth. The total penetration depth of the cone into the HPM was 4 mm. Thus, the raw data recorded by the balance was the negative weight due to the opposing force to penetration of the cone vs. time. The total time of the penetration experiments was 30 s.

## 3. Results and Discussion

### 3.1. Preparation via PEmPTech and Characterization of Polymer Microspheres

**Figure 1:**
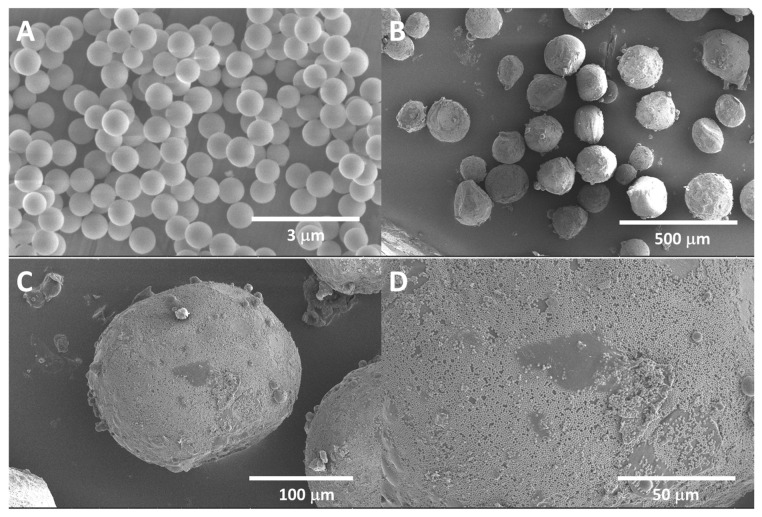
SEM images of (**A**) silica nanoparticles functionalized with glycidyl, (**B**) polymer microspheres obtained via PEmPTech, exhibiting nanostructured surface (**C**,**D**) due to trapping at the oil/water interface of a self-assembled monolayer of silica nanoparticles, the Pickering emulsion stabilizing nanoparticles.

Pickering emulsion polymerization technology (PEmPTech) is a recently developed green, water-based method, also developed by our group and by others [24,25], for the facile synthesis of polymer microspheres with nanostructured surfaces. The method utilizes oil-in-water (*o*/*w*) Pickering emulsions, emulsions that are stabilized by silica nanoparticles, and the dispersed phase is a water-immiscible vinyl-bearing monomer. As a side note, other groups utilize the same technology for producing microporous polymer monoliths [10] and asymmetrically structured Janus membranes [26]. The silica nanoparticles utilized in the current work are 500 ± 10 nm diameter silica nanoparticles modified with glycidyl functional groups on the surface (see Figure 1A), which have been previously shown to produce preferentially *o*/*w* emulsions [27]. The Pickering emulsions stabilization mechanism by nanoparticles has been previously described, and it is mainly due to the interfacial adsorption of the silica nanoparticles at the oil–water interface with the formation of a self-assembled monolayer which acts like a shield preventing the coalescence of the oil droplets [28].

We have prepared *o*/*w* Pickering emulsion, where the oil phase contains MA and 4-VP, DVB, and a common water-immiscible solvent, toluene. The polymerization mechanism resembles suspension polymerization. Interestingly, the PEmPTech is extremely versatile, allowing for a broad spectrum of monomer composition cocktails to be used; for example, partially water-immiscible vinyl-bearing monomers, as well as completely water-insoluble monomers, can be used if they have a common solvent or they are miscible with one another. Upon polymerization of the *o*/*w* Pickering emulsion, the dispersed phase, the oil droplets are converted into [25] poly(4-vinyl pyridine-co-methacrylic acid) polymer microspheres (see Figure 1B,C), whereas the self-assembled monolayer is now trapped and gives the microspheres the typical nanostructuring (see Figure 1D), as observed for the microspheres produced by this technology (PEmPTech). The implications of nanostructuring in the water-wetting of the polymer microspheres are significant. We have previously demonstrated that for a hydrophobic polymer whose typical water contact angles are around 80°, due to the nanostructuring from a self-assembled monolayer of NP-Gly on the surface of the polymer, the wettability increases significantly, lowering the water contact angles to values below 60° characteristic for a hydrophilic surface [26]. This improvement in water wettability due to nanostructuring has significant implications for the extraction of metal ions from aqueous solutions by these polymer microspheres.

**Figure 2:**
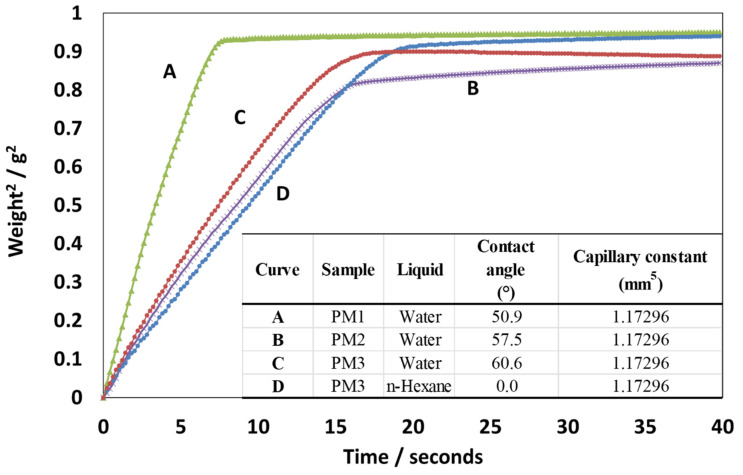
Graph of the liquid intake weight squared vs. time by the capillary packed with powder consisting of polymer microspheres (PMs). The curve (A) represents batch PM3 wetted by the hexane, from which the packing constant C was calculated (see Equation (3)). Curves (B), (C), and (D) represent the water intake with time by the powder consisting of PMs manufactured in three different batches, from which the water contact value was calculated (PM1, PM2, and PM3, respectively). Note that a steeper slope of liquid intake corresponds to a lower contact angle with the liquid. The table inset shows the value of the contact angles with the liquid obtained for each corresponding curve and PM batch with the corresponding capillary constant.

In the current case, for the polymer microspheres synthesized, we have measured the water wettability of the polymer microsphere powder using the Washburn method [29], which is based on monitoring the weight of the liquid intake of a powder due to capillary forces at constant temperature:

(3) $$\cos\theta =\frac{\mu }{C\cdot \rho_{Liquid}^{2}\cdot \gamma_{Liquid}}\cdot \frac{m}{t}$$

where *θ* is the contact angle (wettability); *μ* is the viscosity of the contacting liquid; *ρ* is the density; *γ* is the surface tension; *m* is the mass intake of the liquid; *t* is the time; and *C* is the packing constant dependent on the capillarity, with the capillaries being formed between the powder grains or nanoparticles. In the current case, the capillary constant for the polymer microspheres was determined to be *C* = 1.179 mm^5^ with hexane (see Figure 2).

From the capillary rise data presented in Figure 2, we can see that the water wettability of powder consisting of PMs from three different batches is consistent and is distributed around an average value of the contact angle of 56° ± 5°. Based on this value, we can draw the conclusion that the PMs can be dispersed reasonably well into an aqueous system, such as the PVA solution, for the generation of the HPM composites.

The interaction of the poly(4-vinyl pyridine-co-methacrylic acid) PM with the metal ion can be evidenced from the FTIR-ATR spectra of the PMs before and after the adsorption of Cu(II) ions. Figure S2 shows the IR spectra of the 4-VP-co-MA copolymer—Cu(II) complex. The absorption bands at 1606 (C=N stretching), 1541, 1463, and 1398 cm^−1^ are assigned to the characteristic vibration of the pyridine ring [30,31,32]. The absorption bands at 1076 and 948 cm^−1^ could be assigned to the in-plane and out-of-plane C–H bending of the pyridine ring [30], while the vibration at 1163 and 1076 cm^−1^ could be assigned to the single-bond C-O stretch in the carboxyl. The effect of the Cu(II) adsorption by the PMs can be best observed in the region 1200–1800 cm^−1^. For example, the peak at 1614 cm^−1^ is a new peak, strongly enhanced in the presence of Cu(II) ions and representing the fraction of the coordinated vinyl pyridine rings, i.e., ascribed to the pyridine ring–Cu^2+^ bond vibration [30,32]. On the other hand, after Cu(II) coordination, the characteristic vibrations of the pyridine ring mentioned are blue-shifted to 1517, 1452, and 1384 cm^−1^ [30]. At the same time, the peak at 1705 cm^−1^, corresponding to the stretching modes of the carbonyl groups, is strongly enhanced in the presence of the Cu(II) ions, presumably due to complexation [33], or as Lee et al. ascribe, a liberation of the carbonyl stretching due to the complexation of the acid hydroxyl group [31]. The characteristic stretching vibration for the carboxylate group, usually at 1600 cm^−1^, overlaps with the characteristic bands of pyridine; therefore, an enhancement of the band at 1600 cm^−1^ can be coming from both units, due to pyridinium coordination with the Cu(II) as well as the electrostatic interaction of the carboxylic group with the Cu(II) [32,33]. Thus, the data indicate strong interaction and coordination of the Cu(II) ions by the PMs.

### 3.2. Preparation, Characterization, and Morphology of HPM Composites

In this work, a series of HPM composites have been prepared, where both their compositions vary in terms of microparticle content and preparation method. For example, two categories of HPM composites have been created: (i) simple HPMs with increasing amounts of microsphere content HAM-1 and HAM-2 and (ii) foamed HPM series FAM-1 and FAM-2 with increasing amounts of polymer microspheres that are capable of floating on the water surface (see Table S1).

**Figure 3:**
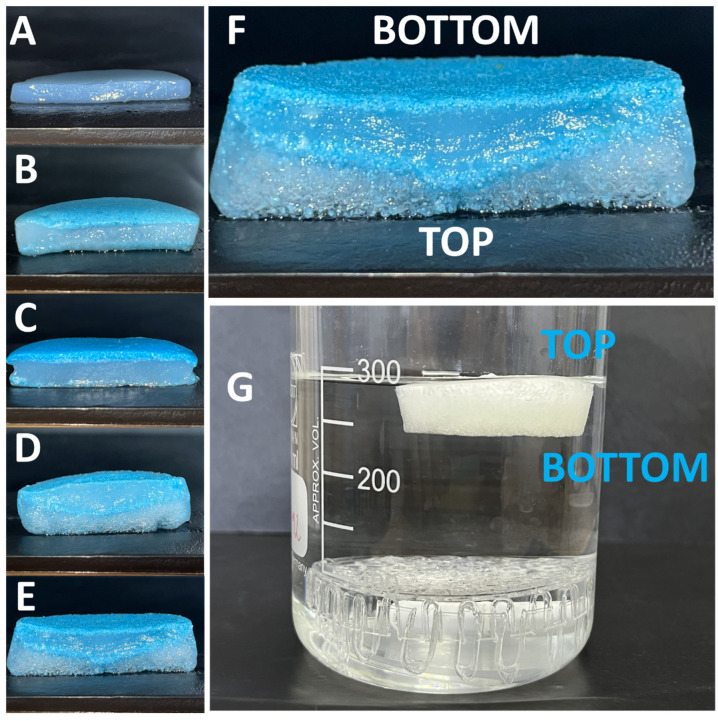
Photographs of the cross-section of the PVA hydrogel (**A**) and of HPM composites (**B**) HAM-1, (**C**) HAM-2, (**D**) FAM-1, and (**E**) FAM-2, having all the same orientation with the bottom of the HPM, upwards, and the top, downwards. The images were taken after HPM composite exposure to a solution of 5 × 10^−2^ M CuCl_2_·2H_2_O. The reference of top and down for the HPM is taken as the position in which the HPM sat during the preparation (gelation). Image (**F**) is a close-up photograph of FAM-2 and (**G**) of the floating FAM-2 HPM composite on the surface of the water.

Photographs of the obtained HPMs and the PVA hydrogel are given in Figure S3 in the Supplementary Materials (SM). From these images (Figure S3), the physical dimensions of the HPMs vary as a function of composition. Although they were prepared in a silicon form that had a diameter of 40 mm, the PVA hydrogel containing no microspheres shrunk by a few millimeters after preparation as compared to the container. The foamed and non-foamed HPM composite FAM-1 and HAM-1 have retained the lateral dimensions of the container, but each has a different thickness, albeit the composition stays the same. The fact that FAM-1 and FAM-2 are thicker than HAM-1 and HAM-2, respectively, is due to the foaming of the former sample. The foaming in the case of the former sample was preserved by instantaneous freezing with liquid nitrogen of the hydrogel after mechanical agitation (shaking). Thus, we can note a difference in morphology between the HAM- and FAM-type samples, while the PVA hydrogel samples are compact and homogeneous. The HAM- and FAM-type samples are heterogeneous, and this was evidenced by cutting the hydrogels and imaging them in the cross-section after the adsorption of Cu(II) ions. Thus, it can be seen from Figure 3 that the HAM-1 and HAM-2 samples are only colored in intense blue in the bottom part of the sample (see Figure 3B,C for the bottom layer), where the polymer microsphere adsorbents have accumulated due to sedimentation during the gelation time. This is explained by the fact that only the PMs are capable of chemically binding Cu(II) ions, while the gel itself can only weakly physically adsorb these ions; thus, the middle of the sample remains white, as with the PVA hydrogel Figure 3A.

The FAM-1 and FAM-2 HPM composite samples (Figure 3D,E) appear structured similar to a sponge, are more voluminous than the HAM-type samples, and the adsorption of Cu(II) evidences a heterogeneous distribution of PM, mostly in the bottom part of the sample, as it can be clearly seen in Figure 3F. The sponge-like structure of the FAM-type sample endows them with the capacity to float on the surface of the water (see Figure 3G), while the HAM-type samples are not capable of floating. This capability is the property we were looking for in HPM composite that could be deployed on the surface of the water to extract metal ions from waters for hydrological mining and provide a valuable secondary source for raw materials, such as metal ions.

**Figure 4:**
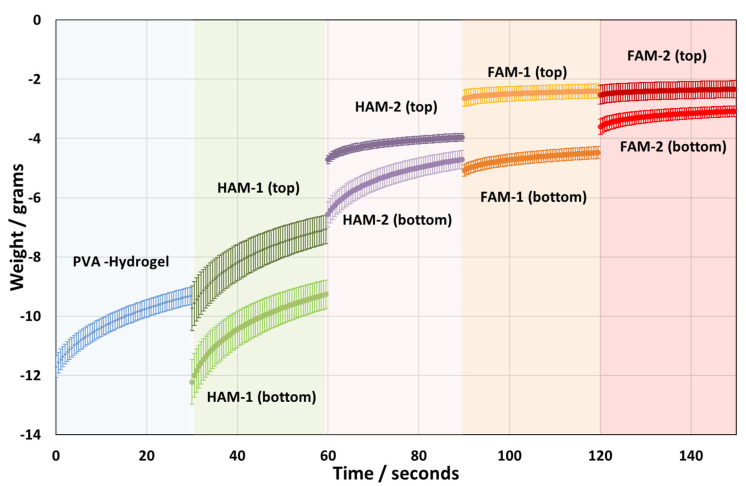
Resistance weight of the HPM to cone penetration vs. time for the series of HPM. The penetration experiment spanned the duration of 30 s for an immersion depth of the cone of 4 mm into the sample. The penetration tests were executed both to the top and to the bottom of the samples. The increase in softness of the material is evidenced by a lower absolute weight resistance to cone penetration. The error bars represent the standard deviation from at least three measurements.

Further, the softness of the HPM composites was evaluated for the top and bottom of the samples using the penetration probe, as described in the Materials and Methods section. The experimental results are presented in Figure 4. Also, penetration experiments were carried out both at the top and at the bottom of the sample to see if there were differences. From the penetration experimental data, we observe three major trends, namely (i) the increase in the softness of the material with the addition and an increasing amount of polymer microparticles added in the HPM composition; (ii) the samples with microparticles are anisotropic, they are softer on the top than on the bottom; and (iii) the foamed samples are softer than the non-foamed samples. For the first case, (i) the addition of polymer microparticles appears to break the cohesion of the hydrogel and soften it considerably; the softness increases in the order PVA-Hydrogel < HAM-1 < HAM-2 < FAM-1 < FAM-2 (see Figure 4). In addition, it is also obvious that the addition of polymer microparticles causes the volume of the sample to change considerably, compared with the dimensions of the reference PVA-Hydrogel sample with the HAM-1, where the reference sample PVA-Hydrogel seems rather hard (Figure 4) and compact (Figure S3). This change in volume from PVA-Hydrogel to HAM-1 can be attributed to the formation of foam during shaking, whereas particles are known to stabilize foams. The increase in the degree of softness from HAM-1 to HAM-2, which differ only in the number of microspheres, appears to support the hypothesis that additional microspheres cause more foam bubbles. For the second case (ii), anisotropy of the sample arises during the gelation process, whereas the microparticles sediment on the bottom part of the sample, while some degree of foam is preserved on top. For the last case, (iii) the freezing with liquid N_2_ of the freshly shaken sample preserves the foam bubbles, while clearly the microspheres sediment on the bottom of the sample. The degree of softness of the FAM-1 and FAM-2 at the top is comparable, while FAM-1 is harder than FAM-2. This is probably due to variations in sample preparation and particle sedimentation.

### 3.3. Role of Morphology of the HPM Composites in Cu(II) Adsorption and Water-Floating Ability

The HPMs were further employed in Cu(II) ion adsorption studies. The experimental procedures for adsorption/extraction of Cu(II) from model water samples with a 5 × 10^−2^ M concentration were kept the same for the control sample, the PVA hydrogel, the HAM-1 and -2, and the FAM-1 and -2 samples, as described in the experimental procedures. Similarly, the Cu(II) ion desorption/recovery studies were kept the same for all samples and were proceeded by treatment with a 5% HCl solution for a period of 12 h, after which the concentration of the Cu(II) ion recovered from the material was measured. Both types of experiments were carried out under gentle stirring of 200 rpm. It can be noted that FAM-1 and FAM-2 samples were floating on the surface of the water (see Figure 3G) both during extraction and during metal ion recovery experiments. The PVA, HAM-1, and HAM-2 samples stayed submerged in the water, at the bottom, all the time; thus, in the case of these samples, an enclosing plastic cage with holes to allow water diffusion was manufactured to isolate the stirrer and avoid the magnetic stirrer physically hitting the sample.

**Figure 5:**
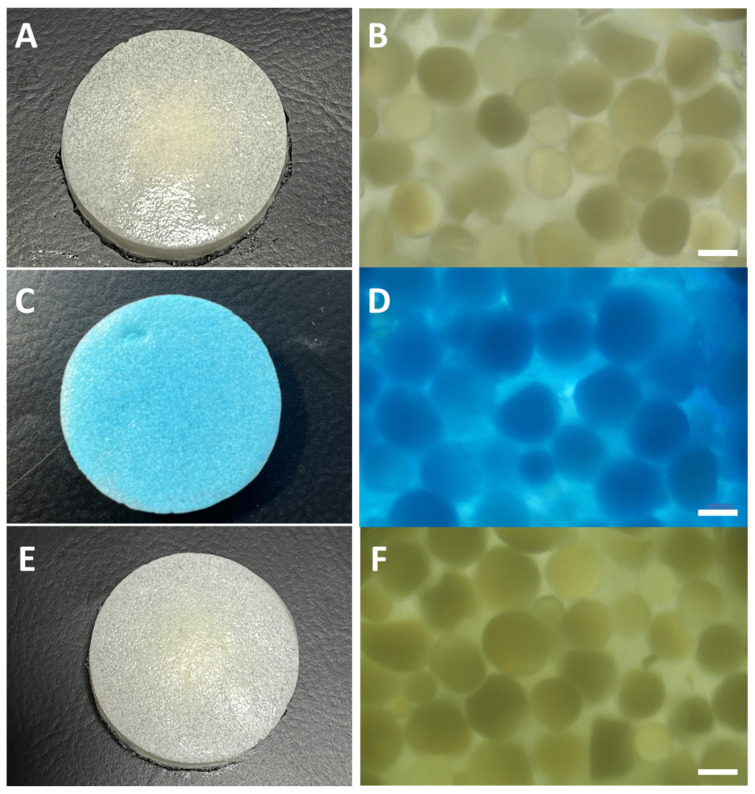
(**A**) Photograph of the as-prepared HPM composite, HAM-2, with the corresponding optical microscope image (**B**) taken with a 10× magnification showing the embedded polymer microparticles. (**C**) Photograph of the same hydrogel after exposure for 12 h to a 5 × 10^−2^ M CuCl_2_·2H_2_O solution and the corresponding optical microscope image (**D**) at 10× magnification, showing a strong blue coloration of the microspheres. (**E**) Photograph of the same hydrogel after being kept in a 5% solution of HCl solution and the corresponding microscope (**F**) at 10× showing a discoloration of the microspheres. The scale bar in the microscope images is 100 μm.

The HAM-1 and HAM-2 samples had a drastic change in color, from white to deep blue, upon absorption of the Cu(II) ions, even compared to the PVA control hydrogel samples (Figure S4). Initially, the HPM composites are white in color, as shown in the image in Figure 5A, and in the optical microscope image (Figure 5A), it can be seen that the embedded polymer microspheres are rather colorless. When the HPM has been exposed for 12 h to a 5 × 10^−2^ M CuCl_2_·2H_2_O solution, its color changes to deep blue (Figure 5C), and the microspheres become intensely blue-colored (Figure 5D). Upon removal from the Cu(II) ion solution and treatment with a 5% solution of HCl, the HAM-2 changes color again (Figure 5E), and the microspheres become colorless, as shown in the optical microscope images in Figure 5F. The same is true for FAM-1 and FAM-2 HPM composites (see Figure S5). The difference in morphology between the HAM- and FAM-type samples noted above is becoming evident after the adsorption of Cu(II) ions. Upon Cu(II) ion adsorption, the HAM-type non-floating submersible samples and the FAM-type floating samples are colored in the bottom part of the sample, where the polymer microsphere mostly accumulated due to sedimentation during preparation (Figure 3B–E).

Thus, due to their unique morphology, the FAM-type samples are capable of floating due to the existence of air bubbles in the foamed part of the sample. However, in contrast to the HAM-type samples, the FAM-type samples show, at least qualitatively, adsorption in the bottom part of the samples, in other words, in the part of the sample with fewer bubbles, where the polymer microsphere adsorbents are concentrated. Next, we will analyze in quantitative terms how these two types of samples perform with the given difference in morphologies and the same composition.

### 3.4. Capacity of Ion Extraction and Recovery of HPM Composites

**Figure 6:**
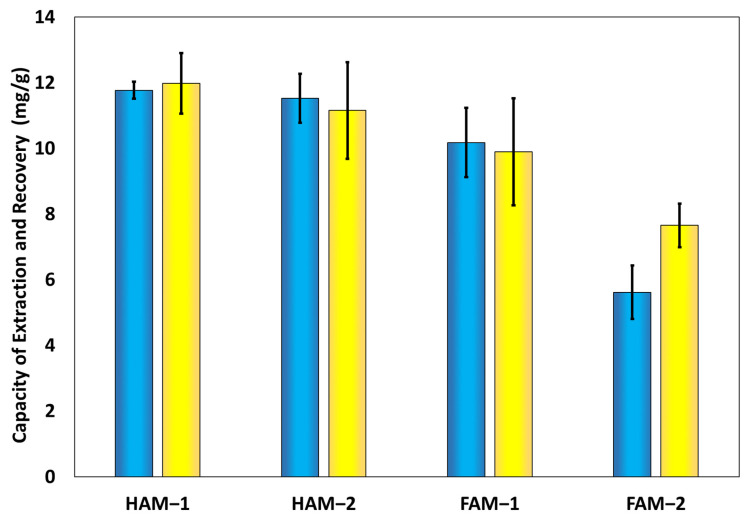
Histogram showing the effective recovery capacity *q_r_* (blue bars) and extraction capacity *q_e_* (yellow bars) data for the HAM- and FAM-type samples, from which the corresponding capacities of the control PVA hydrogel sample have been extracted.

The capacity for Cu(II) ion extraction *q_e_* refers to the adsorption or removal of metal ions from model water samples, stock solutions of 5 × 10^−2^ M concentration. However, because for these materials, we are also interested in the ability to recover the metal ions from the material, we have also measured the Cu(II) recovery capacity *q_r_*, which was achieved by treating the HPM composites with an acidic solution of 5% HCl. This could demonstrate that these materials are indeed feasible to be deployed in technologies interested in recovering raw materials by hydrological mining. Thus, each sample from both classes of composites, HAM type and FAM type, as well as control samples, the polymer microsphere adsorbents and PVA hydrogel control samples, have been employed in at least four cycles of extraction and recovery of Cu(II) ions. The results are presented in Figure 6. Here, we note that the adsorption capacities calculated with Equations (1) and (2) were performed for the entire mass of the hydrogel, and Figure 6 shows the effective *q_r_* and *q_e_* capacities, meaning that the adsorption capacity of the PVA control sample, *q_r_* = 15.7 mg/g and *q_e_* = 11.6 mg/g, accounting for physical adsorption of Cu(II) ion, have been subtracted from each value. From the results presented, it can be immediately noted that the capacities of the HAM-type samples are only slightly less than that of FAM-type samples. Furthermore, while HAM-2 and FAM-2 both contain a double amount of polymer microsphere adsorbents, no significant change in the adsorption capacity within the experimental error for HAM-2 is observed; both the *q_e_* and *q_r_* are comparable to those of HAM-1, while for FAM-2, both the *q_e_* and *q_r_* are slightly less than that of FAM-1. Further, the only parameter that changes between the two sets of samples, HAM and FAM, is their morphology; thus, the effect of the morphology on the adsorption capacity can be understood by comparing HAM-1 to FAM-1 and HAM-2 to FAM-2. Here, we note a decrease in the adsorption performance for the Cu(II) ions of the FAM-2 type samples (*q_e_* = 7.7 mg/g and *q_r_* = 5.6 mg/g) compared to the HAM-2 (*q_e_* = 11.1 mg/g and *q_r_* = 11.5 mg/g) samples of about 31% for *q_e_* and 51% for *q_r_*, due to their unique morphology, whereas we hypothesize that the part containing air bubbles causing their floating ability, contributes less to ion adsorption. Also, a slight change in adsorption capacity can be noted for the HAM-1 (*q_e_* = 12.0 mg/g and *q_r_* = 11.8 mg/g) vs. FAM-1 (*q_e_* = 9.9 mg/g and *q_r_* = 11.2 mg/g) samples of about 18% for *q_e_* and 5% for *q_r_*, we believe also due to their different morphology. By a more careful analysis, however, it can be noted that the adsorption capacity data in Figure 6 are inversely correlated with the softness of the sample in Figure 4. In other words, the softer the sample, which is equivalent to saying the more foamed the sample is, the less adsorption capacity for Cu(II) ions. On the other hand, only the foamed samples are capable of floating on water. Thus, we conclude the tradeoff for having water-floating adsorbent samples is only a slight decrease in the adsorption capacity on the part of the FAM-1 and FAM-2 samples.

**Figure 7:**
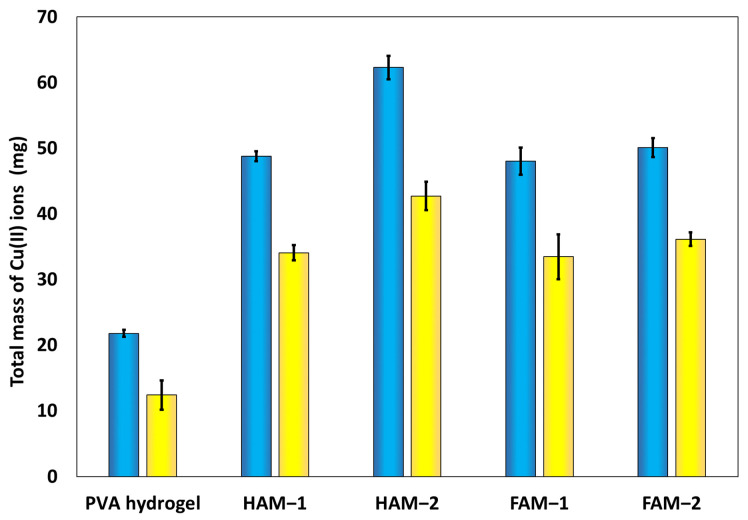
Mass of Cu(II) ions recovered (blue) and extracted (yellow) by the HPM composites.

In absolute terms, while the difference in adsorption capacity of HAM- and FAM-type materials with the same chemical composition is only a reflection of material morphology, the total mass of the Cu(II) ions adsorbed by each HPM composite is due to their chemical composition. The total mass of Cu(II) ions adsorbed by HPM composite series and the reference PVA hydrogel is shown in Figure 7, where a clear change in the total mass of adsorbed metal ions can be seen with the load in the PM adsorbents in the HPM, decreasing in the order HAM-2 (*q_e_* = 42.70 mg/g and *q_r_* = 62.3 mg/g) > FAM-2 (*q_e_* = 36.12 mg/g and *q_r_* = 50.1 mg/g) > HAM-1 (*q_e_* = 34.05 mg/g and *q_r_* = 48.8 mg/g) > FAM-1 (*q_e_* = 33.44 mg/g and *q_r_* = 48.0 mg/g). HAM-2 and FAM-2 have the same amount of polymer microspheres, of 1 g, while the HAM-1 and FAM-1 each have an amount of 0.45 g of polymer microsphere load. Thus, the total mass of adsorbed Cu(II) ions reflects the load of the HPM composite with the PM adsorbent. It is important to note that for FAM-1 and FAM-2 samples, there were no negative effects on their floating capability observed with the PM loading amount.

**Figure 8:**
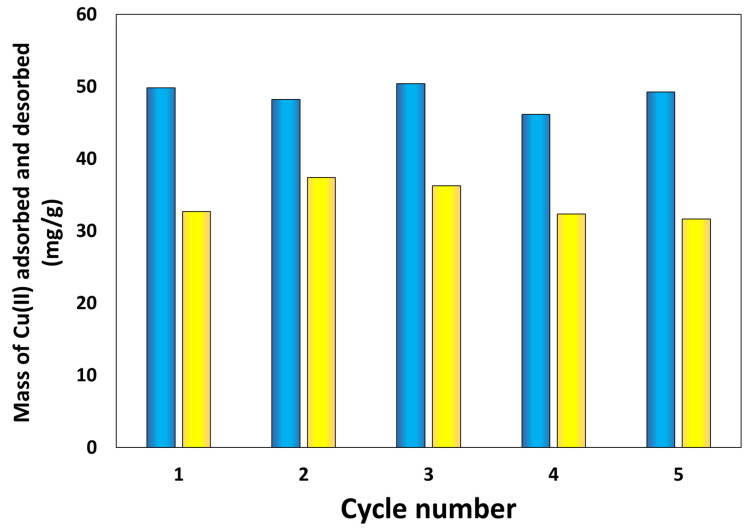
Mass of Cu(II) recovered (blue) and extracted (yellow) by HAM-1 with the cycle number, showing no loss in adsorption and desorption capacities.

In addition, the *q_e_* and *q_r_* capacities have been monitored with the metal ion extraction and recovery cycle number, as with HAM-1, for example (see Figure 8). The mass of Cu(II) ions adsorbed or desorbed from the HPM composite shows no change or loss in capacity up to the fifth cycle of extraction and recovery. These data strongly indicate that these HPM composite materials, especially those of FAM-type morphology, are a new technology that can be successfully deployed on the surface of the water in extraction and recovery of the metal ions from various water sources, lakes, oceans, ponds, etc., for hydrological mining of technological relevant metal ions.

At this point, it is important to compare the current results with the other results in literature. Functional hydrogels generated from polymers with functionality, such as amine, amide functional groups, or carboxylic groups, capable of binding metal ions exhibit excellent adsorption capacities for Cu(II) ions. This should obviously be due to the high functional group density provided by these polymer chains. For example, for the recently reported hydrogels obtained from poly(acrylic acid-co-acrylamide), the Cu(II) ion adsorption capacity was 211.7 mg/g [34], and for the poly(acrylamide-co-sodium methacrylate) hydrogel the Cu(II) ion adsorption capacity was 24.05 mg/g [35]. The former is significantly larger than the one reported here, while the *q_e_* of the latter is comparable to that of the HPM composites. On the other hand, chitosan hydrogel beads, with chitosan being a well-known natural polymer with a high density of glucosamine groups capable of binding metal ions, have a *q_e_* of 130 mg/g. Finally, hydrogel–particle composites, where both particles and the hydrogel are capable of binding the Cu(II) ions, such as hydrogel–clay nanocomposites, have shown a *q_e_* of 68 mg/g [36], while the hydrogel–graphene oxide composite has shown a much-reduced *q_e_* of 5.99 mg/g for Cu(II) ions [37]. Thus, we conclude that the Cu(II) ion adsorption capacities obtained in the current work, *q_e_* ranging from 7.7 to 12.0 mg/g, fall in the middle of the high and low range of the values reported in the literature.

**Figure 9:**
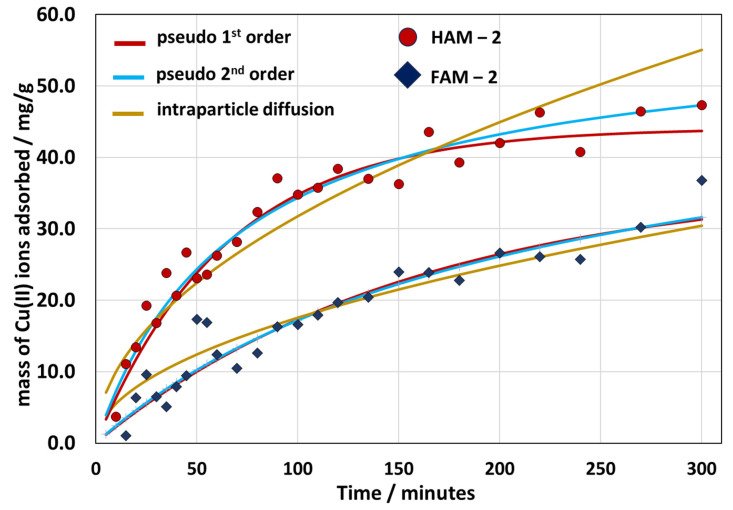
Experimental data of the mass intake of the Cu(II) ions by HAM-2 and FAM-2 with time. Each data set was fitted to a pseudo-first-order kinetic equation, a pseudo-second-order kinetic equation, and an intraparticle diffusion model equation, as indicated in the legend of the graph.

In addition, we have studied the adsorption kinetics of the HAM-2 and FAM-2 materials, the data presented in Figure 9, and we fitted the adsorption data to three different adsorption kinetic models: (i) pseudo-first-order kinetics, described by the equation $q\left(t\right)=q_{e}\left(1-e^{-k_{1}t}\right)$, where *k*_1_ is the rate constant of the first-order adsorption process, *q(t)* is the amount of metal ion adsorbed at any given time, *t*, and *q_e_* at equilibrium [38]; (ii) pseudo-second-order rate expression $q\left(t\right)=\frac{q_{e}^{2}k_{2}t}{1+q_{e}k_{2}t}$, where *k*_1_ is the rate constant of the second-order adsorption process [38]; and (iii) intraparticle diffusion model, which indicates that the intraparticle diffusion is the rate-limiting step $q\left(t\right)=k_{d}t^{0.5}+C$, where the *k_d_* is the intraparticle metal ion diffusion constant, and C is an arbitrary constant [39]. The fit parameters are given in Table S2. It can be seen that the best fit to the data was obtained for the pseudo-second-order kinetics model for HAM-2, followed by the intraparticle diffusion model for FAM-2. This suggests that the pseudo-second-order kinetics model dominates the adsorption characteristic for the HAM-2, and the intraparticle diffusion model is characteristic of the FAM-2 material. In reality, the adsorption phenomenon in the HPM composite cannot be described purely by a single model, but its adsorption characteristic can be best modeled by a combination of different components kinetics of the adsorption and diffusion. Thus, it can be said that FAM-2 has a stronger diffusive component than the HAM-2 composite. Further, judging by the lower value of the rate constants for FAM-2 in Table S2, but also by the fact that the saturation plateau in Cu(II) ion intake is reached much later than that of HAM-2 (see Figure 9), we conclude that the adsorption in FAM-2 is about twice as slow as the adsorption of Cu(II) ions in HAM-2. This can only be explained by the difference in the morphology of the material, whereas the foamed FAM-2 composite lengthens the diffusion path of the Cu(II) ions inside the material to the adsorption sites. Other authors have also related the pseudo-second-order kinetics to chemisorption rather than physisorption [40].

## 4. Conclusions

In this work, we have synthesized HPM composite materials with different morphologies, capable of floating on the surface of water and carrying polymer microsphere adsorbents for Cu(II) metal ions extraction from water samples. The HPMs have shown a good adsorption capacity for these metal ions from water samples, and we have shown that the Cu(II) ions can be easily recovered from these materials. We have demonstrated that the HPMs in different morphologies, such as water-floating FAM types (foamed hydrogels) exhibit only a small loss in the extraction capacity in comparison to the non-foamed hydrogel HAM types, proving the feasibility of these water-floating materials. Further work should focus on loading the floating FAM-type HPM composites with additional amounts of polymer microsphere adsorbents and testing their adsorption performance in complex matrices of ions, samples of different ionic strengths, competitive adsorption studies, and even real marine, lake, or wastewater samples spiked with ions of interest. We believe, in fact, that the HAM and FAM samples have a high technical readiness level to be tested on real water samples. Thus, future work shall also focus on deploying these materials on real water samples either in the laboratory or in a water purification station, at least after the water has been treated with flocculation agents. While these materials are useful in the recovery of Cu(II) metal ions present in the wastewater produced in various industrial activities, such as mining, smelters, foundries, electroplating, batteries manufacturing, etc., it is also conceivable that these could also be deployed in hydro-mining applications. In the hydro-mining applications of water-floating adsorbents, the presence of free copper ions has to be evaluated because the existence of ion species in the given environmental conditions or the presence of bio-produced ligands can lower the concentration of Cu(II) to insignificant levels [41,42]. Nevertheless, areas with excess Cu(II) ions can be identified that produce stress to phytoplankton and aquatic life, and such adsorbents could be involved in environmental remediation and hydro-mining. Further, it may also be of interest to use HAM-type materials in soil remediation applications.

## Data Availability

Data is available at https://osf.io/m8uva/?view_only=4c737cad1ee748dd8922358d5099bca5.

## Supplementary Materials

The following are available online at https://www.mdpi.com/article/10.3390/nano13192619/s1, Figure S1. UV–vis absorption spectrum of 5 × 10^−2^ M CuCl_2_·2H_2_O solution; Figure S2. ATR-FTIR spectra of the PMs before and after adsorption of Cu(II) ions; Figure S3. Photograph of the PVA hydrogel (A), the hydrogel polymer microsphere (HPM) composite HAM-2 (B), and the foamed HPM composite FAM-2 (C); Figure S4. Photograph of the HAM-1 (left) and PVA hydrogel control sample without adsorbent microspheres (right) after being in contact with a stock solution of 5 × 10^−2^ M CuCl_2_·2H_2_O for 12 h, showing a significant change in color from white to deep blue; Figure S5. Photograph of the FAM-2 HPM composites, before and after adsorption of Cu(II) ions from a 5 × 10^−2^ M solution of CuCl_2_·2H_2_O; Table S1. Composition of the HPM composite series in the form of hydrogel and foam; Table S2. The fit parameters of the kinetic adsorption of Cu(II) ions by the HAM-2 and FAM-2 to three different models, as indicated by the equations in the text, and r represents the goodness-of-fit parameter.
