# Supplementary material for: Water-Floating Hydrogel Polymer Microsphere Composites for Application in Hydrological Mining of Cu(II) Ions
Source: Nanomaterials (Basel). 2023 Sep 22;13(19):2619. doi: 10.3390/nano13192619 (PMC10574089; doi:10.3390/nano13192619)
Supplement: Supplementary file 1 [file nanomaterials-13-02619-s001.zip › nanomaterials-2616438-supplementary.pdf]

Supporting Information

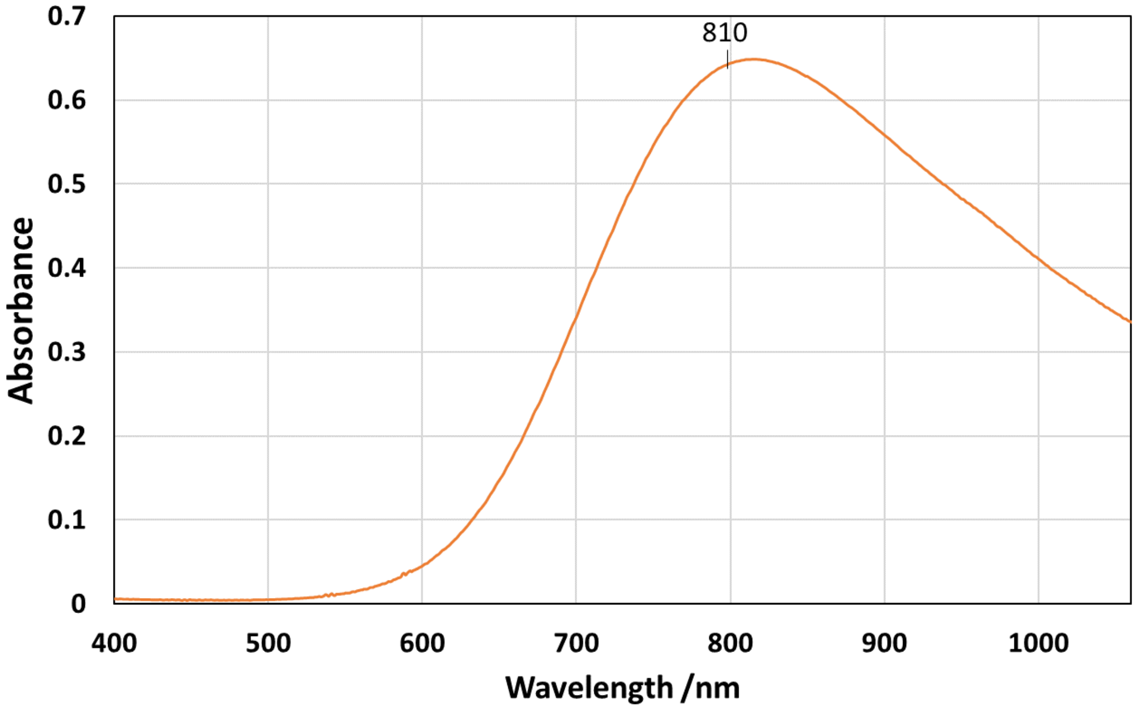

Figure S1. UV-vis absorption spectrum of 5×10^{-2} M $CuCl_2 \cdot 2H_2O$ solution.

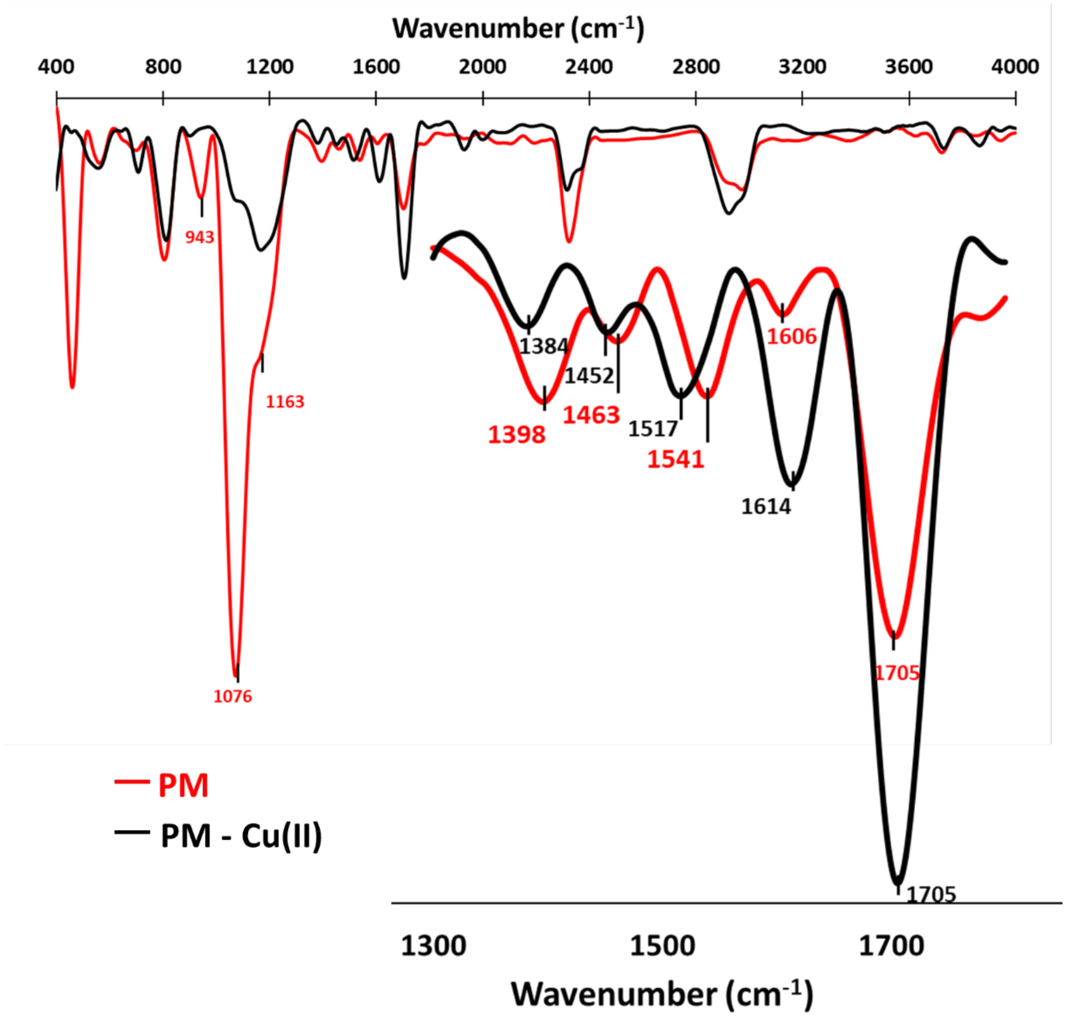

Figure S2. ATR-FTIR spectra of the PMs before and after adsorption of Cu(II) ions.

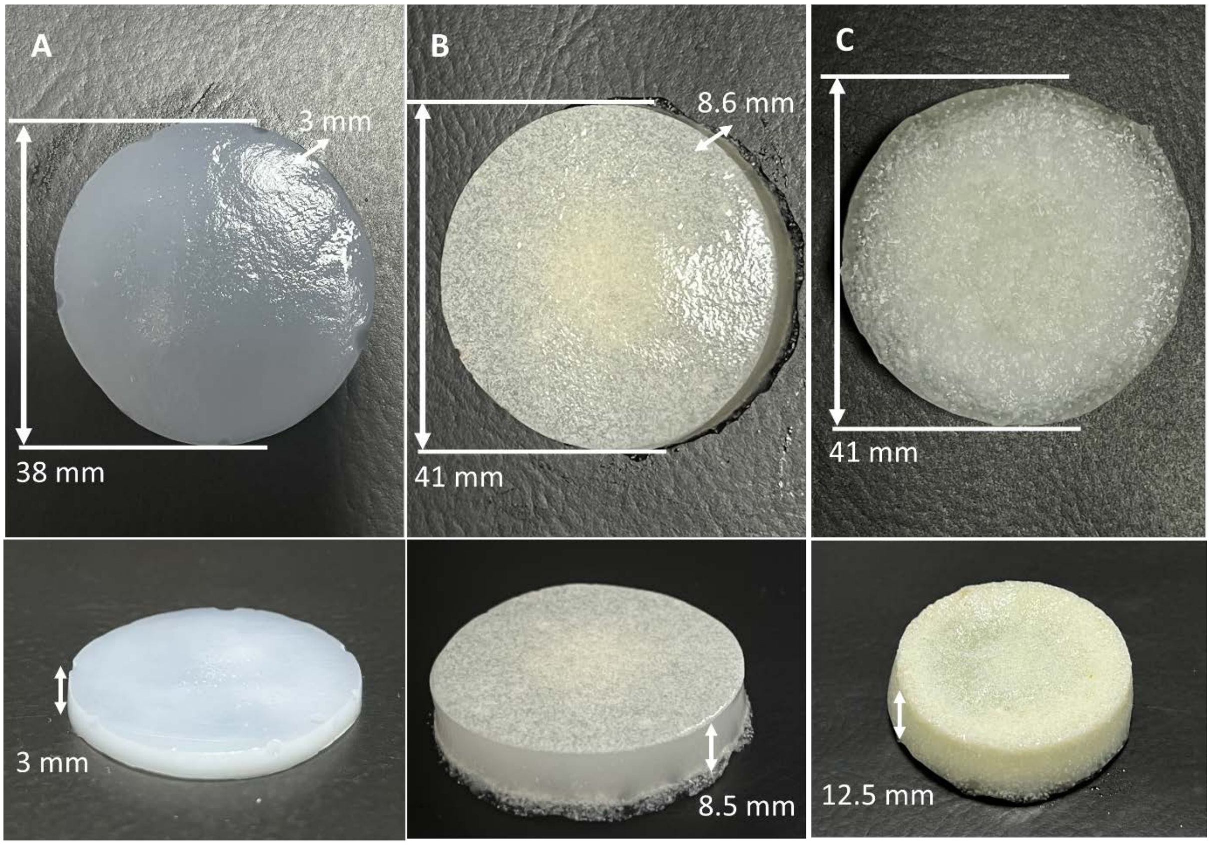

Figure S3. Photograph of the PVA hydrogel (A) and of the hydrogel-polymer microsphere (HPM) composite HAM-2 (B) and of the foamed HPM composite FAM-2 (C).

Table S1. Composition of the HPM composite series in the form of hydrogel and foam.

Sample	PVA (g)	Glycerol (g)	Microspheres (g)	Dry mass (g)	Observations
PVA -Hydrogel	0.45	0.9	-	1.35	hydrogel
HAM-1	0.45	0.9	0.45	1.8	hydrogel
HAM-2	0.45	0.9	1	2.35	hydrogel
FAM-1	0.45	0.9	0.45	1.8	hydrogel - foam
FAM-2	0.45	0.9	1	2.35	hydrogel - foam

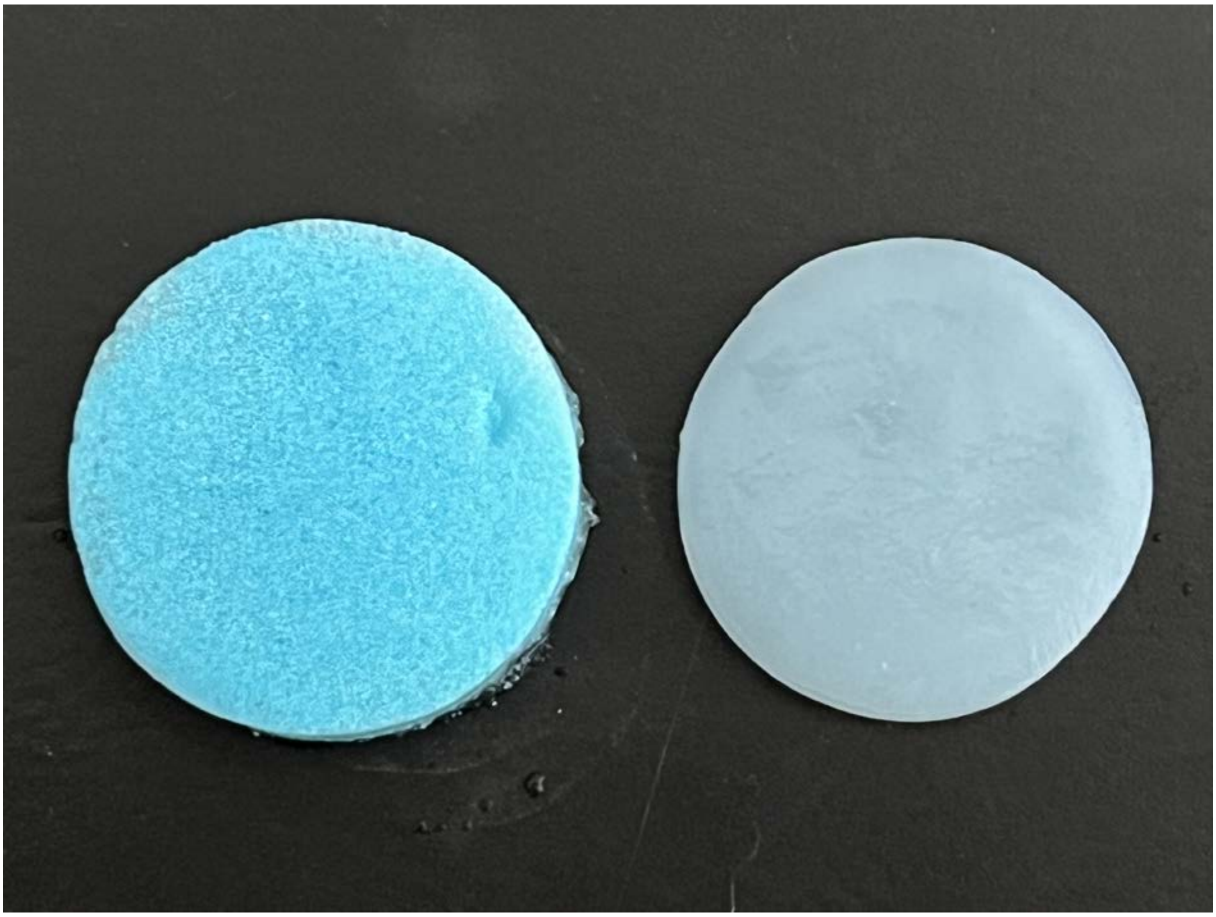

Figure S4. Photograph of the HAM-1 (left) and PVA hydrogel control sample without adsorbent microspheres (right) after being in contact with a stock solution of 5×10^{-2} M $CuCl_2 \cdot 2H_2O$ for 12 hours, showing significant change in color from white to deep blue.

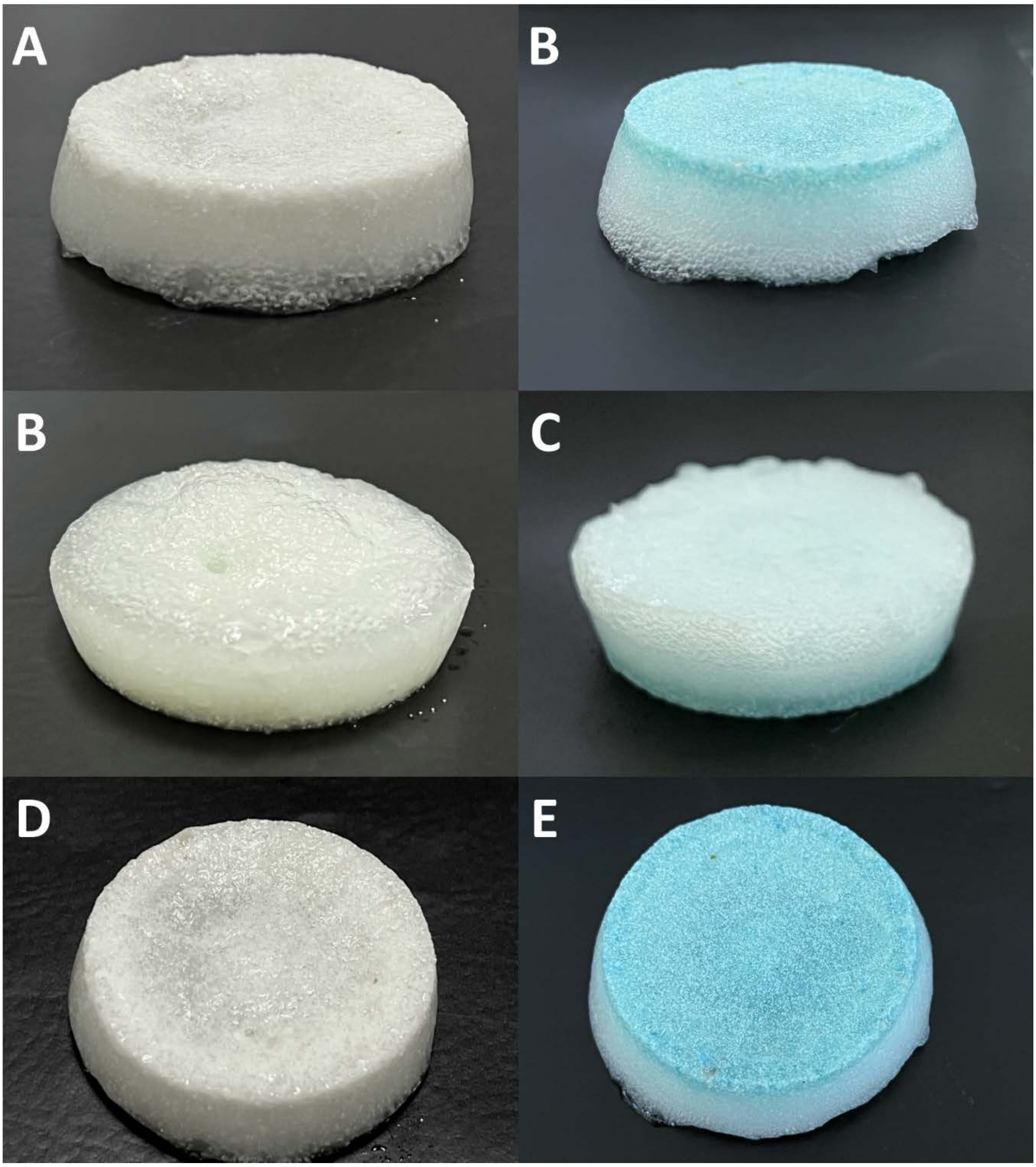

Figure S5. Photograph of the FAM-2 HPM composites, before and after adsorption of Cu(II) ions from a 5×10^{-2} M solution of $CuCl_2 \cdot 2H_2O$.

Table S2. The fit parameters of the kinetic adsorption of Cu(II) ions by the HAM-2 and FAM-2 to three different models, as indicated by the equations in the text and r represents the goodness-of-fit parameter.

Sample	pseudo 1st order			pseudo 2nd order			diffusive model		
	q_e	k_1	r	q_e	k_2	r	k_p	C	r
HAM-2	44.15	0.0155	0.98	58.29	0.0002	0.98	3.18	0.00	0.95
FAM-2	36.87	0.0063	0.95	54.37	0.0001	0.96	1.76	0.00	0.96
